# Amino acid supplementation accelerates resolution of exercise-induced phagocyte infiltration in human skeletal muscle

**DOI:** 10.1080/15502783.2025.2590102

**Published:** 2025-11-30

**Authors:** Mengxin Ye, Giancarlo Condello, Kuo-Ching Chao, Hui-Tai Yang, Chih-Yang Huang, Li-Fan Lai, Luthfia Dewi, Yu-Chieh Liao, Andrew Nicholls, Feng Ji, Nai-Fang Chi, Chia-Hua Kuo

**Affiliations:** a College of Physical Education and Health Sciences, Zhejiang Normal University, Jinhua, People's Republic of China; b Laboratory of Exercise Biochemistry, University of Taipei, Taipei, Taiwan; c Department of Medicine and Surgery, University of Parma, Parma, Italy; d Department of Internal Medicine, China Medical University Hospital Taipei Branch, Taipei, Taiwan; e Cardiovascular and Mitochondrial Related Disease Research Center, Hualien Tzu Chi Hospital, Buddhist Tzu Chi Medical Foundation, Hualien, Taiwan; f Department of Medical Research, China Medical University Hospital, China Medical University, Taichung, Taiwan; g Department of Biotechnology, Asia University, Taichung, Taiwan; h Graduate Institute of Biomedical Science, China Medical University, Taichung City, Taiwan; i Department of Nutrition, Universitas Muhammadiyah Semarang, Semarang, Indonesia; j Soochow University, School of Physical Education and Sports Science, Suzhou, People's Republic of China; k Department of Neurology, School of Medicine, National Yang Ming Chiao Tung University, Taipei, Taiwan; l Department of Neurology, Taipei Veterans General Hospital, Taipei, Taiwan; m Laboratory of Exercise Biochemistry, The Education University of Hong Kong, New Territories, Hong Kong SAR, China

**Keywords:** Neutrophil, MPO, amino acids, *in vivo* mitochondrial transfer, ROS

## Abstract

**Background:**

Amino acids activate neutrophil phagocytosis and free radical release *in vitro*.

**Aim:**

We examined the effects of amino acid supplementation on post-exercise accumulation of myeloperoxidase-positive (MPO⁺) cells in human skeletal muscle using a randomized, double-blind, placebo-controlled crossover design.

**Methods:**

Ten young men (22 ± 2.8 years) consumed either amino acids (15 g) or an isocaloric placebo before resistance exercise. Biopsies of the vastus lateralis muscle were collected at baseline, immediately after exercise (0 h), and 24 h post-exercise.

**Results:**

Resistance exercise increased MPO⁺ cell infiltration (+161%, *p* = 0.02) and 8-hydroxy−2-deoxyguanosine (8-OHdG) levels (+66%, *p* = 0.02) at 24 h. Amino acid supplementation accelerated MPO⁺ cell infiltration to 0 h (+100%, p = 0.03), which diminished by 24 h post-exercise (+53%, *p* = 0.06). Immunofluorescence co-staining revealed that MPO⁺ cells exhibited markedly higher mitochondrial density (TOM20-labeled) and integrated with the injured regions of adjacent myofibers showing lower mitochondria. Other infiltrating MPO-negative cells also contributed mitochondria to exercised muscle tissue, resulting in an overall ~2-fold increase in mitochondrial content during 24-h recovery (*p* < 0.001), similar under both supplementation conditions. Cellular senescence marker p16Ink4a mRNA decreased by 58% at 24 h post-exercise, with an earlier reduction observed under amino acid treatment (0 h: –49%, *p* = 0.05).

**Conclusion:**

These findings indicate that amino acid supplementation accelerates the resolution of inflammation in exercised human skeletal muscle. Immunofluorescence evidence further suggests that infiltrating bone marrow-derived cells contribute to fast mitochondrial gains as part of the muscle damage-response following exercise.

## 1 Introduction

Resistance exercise inevitably causes muscle fiber disruption, triggering phagocyte infiltration [1,2]. During this inflammatory process, infiltrating immune cells recognize and lyse damaged components of myofibers and dysfunctional cells to lower cellular senescence in the challenged muscle tissue [3]. Cell lysis during the phagocytic phase of inflammation demands immediate cell renewal in muscle tissues, which needs nitrogen source from amino acid or protein for nucleic acid and protein synthesis [4]. Recent *in vitro* studies have shown that amino acid supplementation stimulates neutrophil phagocytic activity and increases reactive oxygen species (ROS) production [5,6], suggesting a nitrogen nutrient-sensing mechanism that regulates cell turnover through inflammation. However, supporting *in vivo* evidence in human skeletal muscle is scarce.

In healthy adults, most phagocytes are produced inside bone marrow and continuously released into circulation due to their short lifespan [7,8]. They are the early wave of immune cells infiltrating into damaged tissue [9]. These phagocytes highly express myeloperoxidase (MPO) [9], a heme-containing enzyme that catalyzes the production of hypochlorous acid (HOCl) and hydroxyl radicals from hydrogen peroxide and chloride ions [10]. The ROS generated in this process contribute to the swift lysis of damaged portion of myofibers or senescent cells in tissue caused by various types of challenges (i.e. physical injury or infection), facilitating cell renewal during tissue repair [11–13]. However, excessive phagocyte mobilization and activation, accompanied by persistently high ROS levels, can lead to unwanted tissue damage [10,13] and bone marrow cell exhaustion [14].

While mitochondria are regarded as a major source of ROS produced during oxidative phosphorylation [15], those in phagocytes exhibit minimal respiratory activity for ATP synthesis. This paradox is explained until recently by several *in vitro* studies, which revealed that mitochondria can transfer between myeloid cells [16,17] and injured myocytes [18]. The mitochondria swapping phenomenon suggests a previously undescribed role for bone marrow cells as a source of fresh mitochondria for replenishing peripheral cells during tissue regeneration. Mitochondrial transfer is an underappreciated mechanism for maintaining metabolic function in myofibers, given their short half-life of only two weeks [19,20]. Since now, no *in vivo* study is currently available to provide physical evidence of interactions between mitochondria-carrying immune cells and myofibers in human skeletal muscle. In this study, we aimed to determine whether MPO^+^ cells constitute mitochondria-rich populations and their engagement to myofibers in human skeletal muscle using immunofluorescence co-staining. We further assess the impact of amino acid supplementation on MPO^+^ cell infiltration in human skeletal muscle following resistance training.

## 2 Materials and methods

### 2.1 Participants

**Figure 1.**
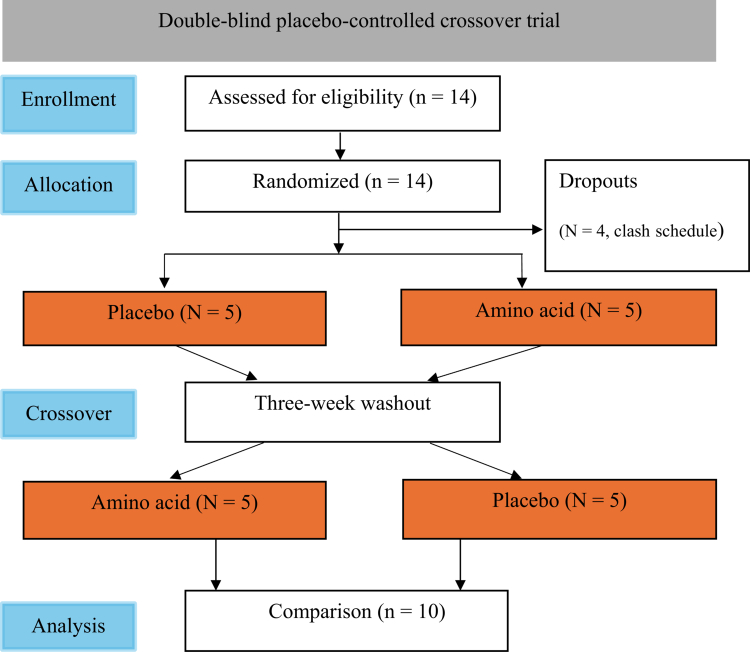
Consort diagram. Flowchart illustrating the study design and participant allocation.

Ethical approval was obtained from the Institutional Review Board of the University of Taipei (Approval No. IRB−2022−044). The study adhered to the principles of the Declaration of Helsinki. Eligible participants were sedentary young men (aged 20−30 years) who exercised fewer than twice per week. Exclusion criteria included smoking, inflammatory or metabolic conditions, and regular exercise training. Initially, 14 candidates were recruited, but four withdrew due to scheduling conflicts during the study. The final cohort consisted of 10 participants (age: 22 ± 2.8 years, height: 172 ± 1.8 cm, weight: 68 ± 1.7 kg, BMI: 23 ± 0.6 kg/m²). Participants were instructed to abstain from exercise and medical/nutraceutical supplements before high-intensity interval exercise (HIIE) during the study period. Written informed consent was obtained from all participants before the study commenced. The Consort diagram is shown in Figure 1.

### 2.2 Experimental design

A double-blind, placebo-controlled, counterbalanced crossover study was conducted to assess the effects of amino acid supplementation on muscle recovery from immune response following an acute bout of resistance exercise. Washout period was >3 weeks, under the assumption that amino acid consumption would not exert a carryover effect during this interval. Muscle biopsies were collected at three time points: baseline (3 weeks before exercise), immediately post-exercise (0 h), and 24 h post-exercise. Participants were instructed to refrain from any form of training for one week prior to both the baseline biopsy and resistance exercise.

### 2.3 Resistance exercise

A certified trainer supervised all exercise sessions to ensure consistency. Participants performed barbell squats using a Smith machine, lowering the barbell in a controlled manner until their knees reached a 90 ° angle and their hips lightly touched an adjustable multi-angle bench. Each session began with an individualized warm-up, followed by 4 sets of barbell squats (8 repetitions per set) at 70% of their one-repetition maximum (1-RM), with 90-second rest intervals between sets, according to a previous study [21].

**1-RM Determination:** Maximum muscle strength (1-RM) was assessed during the first visit. Participants performed dynamic stretching, followed by a structured warm-up: 10 repetitions at 50% of estimated 1-RM; 5 repetitions at 75%, and 1 repetition at 90−95%. After a 5-minute rest, participants attempted progressively heavier lifts (~5% increments) with 3−5 minutes of rest between attempts until 1-RM was determined.

### 2.4 Amino acid supplementation

Participants were randomized and blinded to supplementation. They received a total of 15 g of either amino acids or an isocaloric placebo (maltodextrin) in three separate oral doses before exercise. This dosage is known to enhance muscle protein synthesis [22]. Each dose contained either 5 g of amino acid powder (2 g leucine, 2 g arginine, 0.5 g whey protein, and 0.5 g BCAA) (iMuso, TOP-Pharma, Taichung, Taiwan) or 5 g of maltodextrin. The addition of arginine to the BCAA-whey protein mixture provides substrate for nitric oxide production to satisfy the need for muscle repair [23]. To activate neutrophil function [5,6], we provided amino acid before exercise. Supplements were administered at three time points: 1 h, 30 min, and immediately before exercise. To mask flavor differences, both the amino acid and placebo powders were supplemented with orange flavoring, citrate, and sucralose.

To minimize potential dietary habit on exercise effect, participants were instructed to maintain their usual meals throughout the study and had unrestricted access to water. A registered dietitian conducted a dietary review of meals for three days before the exercise session. To standardize pre-exercise nutrition, participants consumed a 250 kcal isocaloric meal (Ensure; Abbott Nutrition, Lake County, IL, USA) 12 h and 2 h before the exercise challenge.

### 2.5 Muscle micro-biopsy

Muscle micro-biopsies were performed by a certified physician using a 14-G Temno disposable cutting needle (Cardinal Health, McGaw Park, IL, USA) after administering local anesthesia (2% lidocaine). Tissue samples were collected from the vastus lateralis muscle at 3 cm and 20 cm proximal to the patella.

A total of five biopsies were taken: one at baseline (3 weeks before exercise) and two additional biopsies from the contralateral leg at 0 h and 24 h post-exercise. The crossover trial followed the same procedure. Muscle samples were immediately placed into Eppendorf microtubes containing 4% paraformaldehyde (PFA) and later analyzed using immunofluorescence and histochemical staining techniques [3].

### 2.6 Real-time polymerase chain reaction (RT-PCR)

Total RNA was extracted from muscle biopsy samples using the TOOLS EasyPrep Total RNA Kit (BioTools, New Taipei, Taiwan) following the manufacturer’s instructions. Approximately 5 mg of muscle tissue was homogenized on ice using a POLYTRON® system (PT 3100 D, KINEMATICA AG) in 300 μl of denaturation buffer containing guanidine thiocyanate to lyse proteins and release nucleic acids. The extracted RNA was then reverse transcribed into cDNA using the TOOLS Quant II Fast RT Kit (BioTools, New Taipei, Taiwan).

Reverse-transcribed cDNA was amplified by RT-PCR according to the manufacturer’s protocol, with each sample measured in duplicate. The following primer sequences were used for qPCR: p16^INK4a^ mRNA (5′: CCTGTCACTGTCTTGTACCCT, 3′: GGCGTTTGGAGTGGTAGAAATC) and RPP30 mRNA (5′: GGGCCCGGCTCTATGA, 3′: CAGGCCTCGGTCAATCG).

Amplification was performed on a QuantStudio™ 6 Flex Real-Time PCR System (Applied Biosystems, Foster City, CA, USA) using a standard protocol: initial polymerase activation and DNA denaturation at 95 °C for 30 s, followed by 40 cycles of amplification (95 °C for 5 s, 50–65 °C for 30 s). Gene expression levels were quantified using the 2^−ΔΔCt^ method, normalized to the geometric mean of the housekeeping gene (RPP30), and expressed as fold change relative to RPP30.

### 2.7 Immunofluorescence staining

To identify phagocytes, reactive oxygen species (ROS), and mitochondria in muscle cross-sections, triple immunofluorescence staining was performed to detect MPO (staining phagocytes), 8-OHdG (staining DNA oxidation), and TOM20 (staining mitochondria), as follows: 1) MPO (Myeloperoxidase): Rabbit anti-human MPO (ab9535, 1:100, Abcam, Cambridge, UK) with IgG-TAMRA−594 goat anti-rabbit (TAFB02−594, ready to use, BioTnA, Kaohsiung, Taiwan) for red fluorescence; 2) 8-OHdG: Mouse anti-human 8-OHdG (ab48508, 1:100, Abcam, Cambridge, UK) with IgG-FAM−488 goat anti-mouse (TAFB01−488, ready to use, BioTnA, Kaohsiung, Taiwan) for green fluorescence. TOM20 (Mitochondrial Marker): Rabbit anti-human TOMM20 (GTX133756, 1:1000, GeneTex, Hsinchu, Taiwan) with IgG-Cy5−670 goat anti-rabbit (TAFB02−670, ready to use, BioTnA, Kaohsiung, Taiwan) for pink fluorescence. A separate human biopsied muscle sample was used to confirm higher extramyofibrillar location of mitochondria than myofibers by staining with COX4 monoclonal antibody (IB52C31H10), Alexa Fluor™ 488, eBioscience, ThermoFisher (Waltham, MA, USA).

### 2.8 Imaging and quantification

Immunofluorescence staining was assessed by two independent examiners using the same criteria. Results were considered valid if the correlation values exceeded 0.7. Representative images were captured at 20 × magnification using OLYMPUS OlyVIA 3.21 (OLYMPUS, Tokyo, Japan), with scale bars ranging from 200–500 µm. To minimize background interference, brightness was manually adjusted with the same parameters for both independent examiners. ImageJ (National Institutes of Health, Bethesda Maryland, USA) was used to quantify positive staining areas as a percentage of the total area. The minimum size threshold for positive markers (8-OHdG, TOM20, and MPO) was set at 0.785 µm² for consistency.

### 2.9 Statistical analysis

A linear mixed-effects model was used to estimate the fixed effect of treatment and the random effects representing individual variability over time. Power analysis indicated that a minimum of four participants per group would provide 80% power to detect the expected change in mitochondrial content after exercise from baseline, and a minimum of ten participants per group to provide 80% power to detect the expected difference in MPO⁺ cell counts between Amino acid and Placebo conditions, assuming a two-sided alpha of 0.05. Cohen’s *d* was calculated for the resulting effect size from post hoc analysis and interpreted as trivial (0−0.19), small (0.20−0.59), moderate (0.60−1.19), large (1.20−1.99), very large (2.0−4.0), and extremely large effects (>4.0). Data are presented as mean ± standard error (SE), and statistical significance was set at *p* ≤ 0.05.

## 3 Results

### 3.1 MPO^+^ cell infiltration and oxidative DNA damage in skeletal muscle

**Figure 2.**
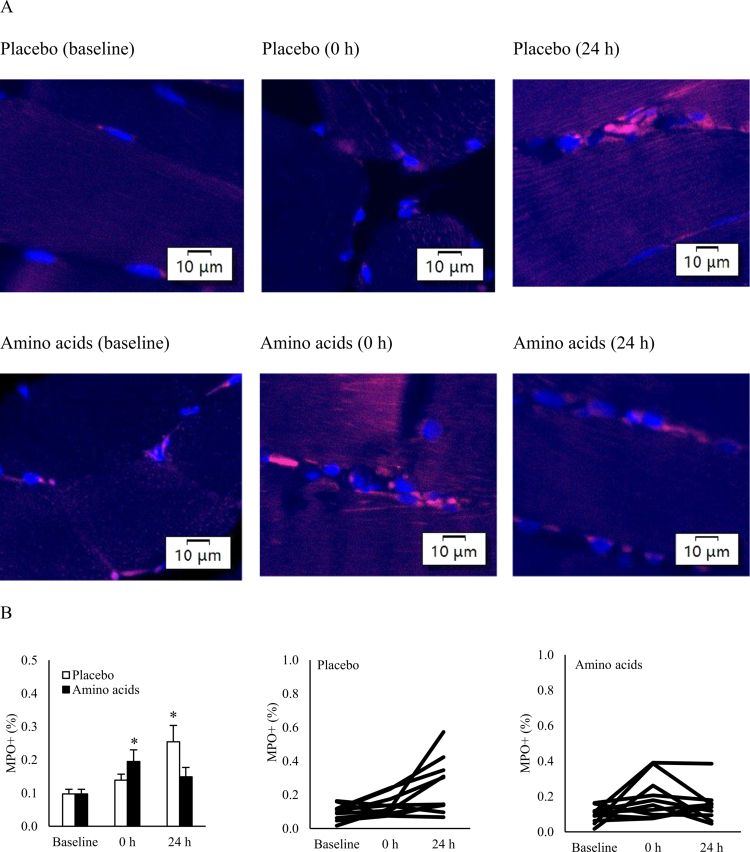
Phagocyte infiltration in human skeletal muscle following resistance exercise. (A) Representative immunofluorescence images showing phagocyte infiltration (labeled by MPO antibody, red) and nuclei (stained with DAPI, blue). (B) Quantification of phagocyte infiltration showing a significant increase 24 h post-exercise. Pre-exercise amino acid supplementation accelerated the resolution of this response to 0 h. *p ≤ 0.05 vs. Baseline. Data are presented as mean ± SE. Abbreviation: MPO, myeloperoxidase.

Resistance exercise led to a progressive increase in phagocyte infiltration, as indicated by MPO^+^ cells in skeletal muscle (Figure 2). MPO^+^ cells increased by 42% (0.097 ± 0.014 to 0.139 ± 0.018% area, *d* = 0.82; *p* = 0.04) immediately after exercise (0 h), further increased to 161% after 24 h above pre-exercise baseline (0.254 ± 0.049% area, *d* = 1.37; *p* = 0.02). Amino acid supplementation accelerated this response, leading to a 100% increase at 0 h (0.195 ± 0.035% area, *p* = 0.02), with levels returning to baseline within 24 h.

**Figure 3.**
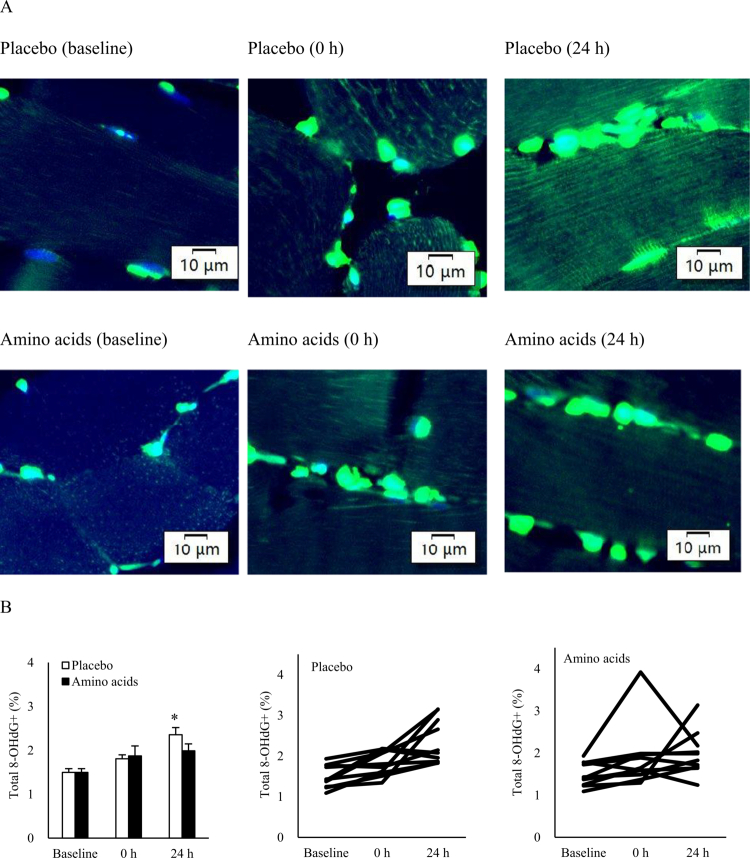
DNA oxidation in human skeletal muscle after resistance exercise. (A) Representative immunofluorescence images showing DNA oxidation (labeled by 8-OHdG, green) and nuclei (stained with DAPI, blue). (B) Overall 8-OHdG levels significantly increased 24 h post-exercise, regardless of whether they originated from nuclear or mitochondrial DNA. No significant changes were observed following pre-exercise amino acid supplementation. *p ≤ 0.05 vs. Baseline. Data are presented as mean ± SE. Abbreviation: 8-OHdG, 8-hydroxy−2'-deoxyguanosine.

**Figure 4.**
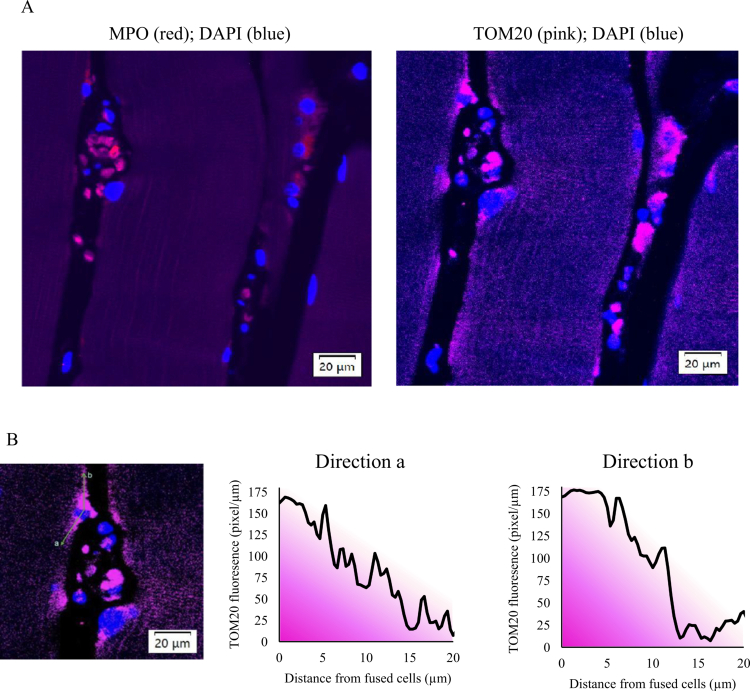
Injured myofibers acquire mitochondria through cell fusion in human skeletal muscle. (A) Myeloperoxidase-expressing cells (left panel: MPO⁺, red) exhibit markedly higher mitochondrial density (right panel: TOM20-labeled, pink) and cluster near damaged myofibers (relatively lower mitochondrial content). (B) Immunofluorescence co-staining of a single myofiber reveals a directional diffusion pattern (right panel), indicating that mitochondria are acquired by the myofiber from fused MPO⁺ cells (left panel).

Similarly, 8-hydroxy−2'-deoxyguanosine (8-OHdG) levels in skeletal muscle increased progressively after resistance exercise, rising by 22% at 0 h and reaching 66% above baseline at 24 h (1.449 ± 0.080 to 2.398 ± 0.177% area, *d* = 2.31; *p* = 0.02) (Figure 3). However, the oxidative DNA damage was minimal when amino acid supplementation was provided. The 8-OHdG signals were predominantly localized in the nucleus and mitochondria, as shown in the stained muscle images from a representative individual (Figures 3 and 4).

### 3.2 Mitochondria-rich cells surrounding damaged myofibers in skeletal muscle

Mitochondrial distribution was assessed using immunofluorescence staining, labelled by TOM20 antibody (Figure 4). Mitochondria-rich cells were predominantly localized at damaged or structurally disrupted portion of myofibers (Figure 4A). Among these, MPO^+^ cells were identified as one of the mitochondrial-rich cell types residing outside myofibers (right panel). Some MPO^+^ cells integrated with myofibers in the damage sites. Other MPO⁻ cells also contributed mitochondria to the myofibers. Single-myofiber imaging revealed a diffusion gradient of mitochondria (pink fluorescence) extending from visually integrated MPO⁺ cells into the myofiber cytoplasm (Figure 4B).

### 3.3 Resistance exercise increased mitochondria in skeletal muscle

**Figure 5.**
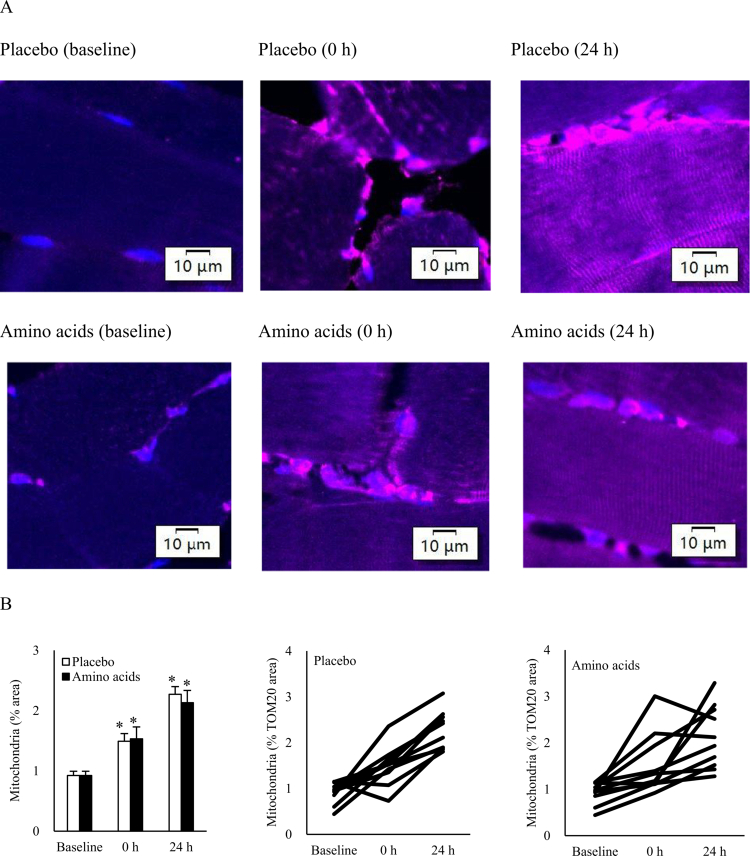
Mitochondrial gains in human skeletal muscle following resistance exercise.(A) Immunofluorescence images from a representative participant showing increased mitochondrial content (TOM20-labeled, pink) in muscle tissues following resistance exercise, similar for both Placebo- and Amino acid- supplemented conditions. Immediately post-exercise (0 h), mitochondria are predominantly localized in cells surrounding the damaged (necrotic) regions of myofibers, with little diffusion into the myofiber cytoplasm, whereas intramyofibrillar mitochondrial content increases at 24 h post-exercise.(B) Mitochondrial gains are primarily driven by the infiltration of mitochondria-rich cells that integrate into the necrotic regions at the myofiber periphery. *p ≤ 0.05 vs. Baseline. Data are presented as mean ± SE.

Figure 5 illustrates total mitochondrial content in muscle tissue after resistance exercise, independent of cell-specific location. A higher concentration of mitochondria was located in extramyofibrillar cells compared to the myofiber cytoplasm (upper panel of Figure 5). These extramyofibrillarly located cells contribute to the overall post-exercise increase in mitochondrial content within muscle tissue. Both the placebo and amino acid supplementation exhibited comparable increases in mitochondrial abundance following resistance training.

Resistance exercise acutely increased overall mitochondrial content in skeletal muscle tissues to a similar extent for placebo- (24 h: 0.923 ± 0.071 to 2.271 ± 0.127% area, *d* = 4.14, *p* < 0.001) and amino acid-supplemented conditions (24 h: 0.923 ± 0.071 to 2.132 ± 0.203% area, *d* = 2.51, *p* < 0.001). This change is mainly contributed by extramyofibrillar mitochondria-dense cells. Intramyofibrallar mitochondria increases was observed 24 h after exercise.

### 3.4 Amino acid supplementation accelerates senolytic effect of exercise in skeletal muscle

**Figure 6.**
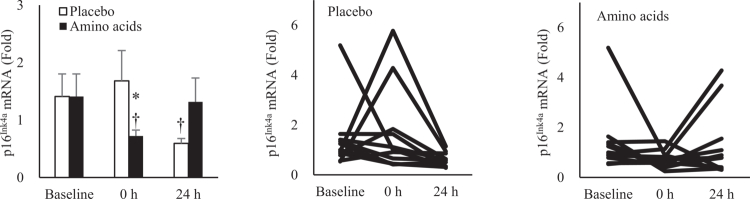
Senolytic effects of resistance exercise in human skeletal muscle enhanced by amino acid supplementation. Cellular senescence marker p16^INK4a^ mRNA in muscle tissues decreased 24 h after resistance exercise. This reduction was accelerated by pre-exercise amino acid supplementation to 0 h. *p ≤ 0.05 vs. Baseline; † *p* ≤ 0.05 vs. Placebo. Data are presented as mean ± SE.

Resistance exercise resulted in decreases in p16^Ink4a^ mRNA expression in human skeletal muscle 24 h post-exercise (*d* = 2.44, -58%, *p* = 0.04). However, when amino acids were supplemented before exercise, p16^Ink4a^ mRNA levels decreased immediately post-exercise and returned to baseline within 24 h (*d* = 1.93, –49%, *p* = 0.05) (Figure 6).

## 4 Discussion

### 4.1 Major findings

Amino acids are known to stimulate phagocytic action of immune cells and induce ROS production *in vitro* [5,6]. In this study, we provides the first *in vivo* evidence that pre-exercise amino acid supplementation advances neutrophil infiltration into skeletal muscle, followed by a more rapid resolution of the immune response induced by resistance exercise. Together with the observed suppression of 8-OHdG accumulation in exercised muscle, these findings suggest that amino acid supplementation shortens the resolution time of exercise-induced muscle inflammation. Furthermore, we observed that MPO⁺ cells contained substantially more mitochondria than the surrounding myofibers. This unexpected observation, together with the directional gradient of mitochondria concentration extending from engaged MPO⁺ cells toward damaged regions of myofiber cytoplasm, implicates active mitochondrial gain by cell fusion. Traditionally, mitochondria within contracting myofibers are thought to be maintained through a balance between mitophagy and biogenesis to sustain ATP synthesis. The findings of the present study introduce a novel perspective, implicating that infiltrating bone marrow-derived cells in exercised human skeletal muscle may also contribute to mitochondrial replenishment as part of the damage response to exercise.

### 4.2 Amino acids accelerate resolution of exercise-induced MPO^+^ cells infiltration in skeletal muscle

Resistance exercise is well-known to induce muscle damage due to eccentric contraction of myofibers [24]. MPO^+^ cell infiltration is an early immune response to muscle damage [9], and plays a crucial role in wound healing in animals [25]. The duration of MPO^+^ cell accumulation in skeletal muscle reflects repair demands of the tissues. In this study, MPO^+^ cell accumulation peaked earlier with pre-exercise amino acid supplementation compared to the placebo condition, suggesting a shortened resolution time of inflammation due to the accelerated immune response.

In the Placebo trial, a progressive increase in 8-OHdG fluorescence indicated sustained ROS release from neutrophilic phagocytosis during the 24-h recovery period [6], whereas this response became insignificant after amino acid supplementation. It is likely contributed by enhanced wound healing capacity of bone marrow-derived neutrophils based on *in vitro* evidence [26]. Myeloperoxidase (MPO), abundantly expressed in neutrophils [9], facilitates phagocytosis and mediates the lysis of damaged portion of myofibers through free radical production in mechanically loaded muscle tissue [27]. In addition to this effect, reduced inflammation time observed with amino acid supplementation may also be linked to enhanced myocyte differentiation, as suggested by a previous animal study [28].

### 4.3 MPO^+^ cells as mitochondrial carriers

The markedly higher mitochondrial content in MPO⁺ cells compared with myofibers is unanticipated, as neutrophils are generally glycolytic [29–31], whereas contracting myofibers require extensive aerobic ATP production. Previous *in vitro* studies have demonstrated that circulating bone marrow-derived cells can donate mitochondria to peripheral recipient cells, thereby restoring metabolic function [32]. Stem cells [32] and platelets [33,34] have been the most studied bone marrow-derived cells as mitochondrial donors. Mitochondrial transfer from MPO^+^ cells remains largely unexplored. The present findings, revealing a mitochondrial diffusion gradient from MPO⁺ cells to the engaged myofiber cytoplasm (Figure 4B), suggest that neutrophils may serve as conduits for mitochondrial replenishment from bone marrow to regenerating peripheral cells following physiological challenges. Notably, the clustering of neutrophils around damaged myofibers indicates that physical damage may serve as the trigger for mitochondrial replenishment from bone marrow-derived cells.

Mitochondria in muscle tissues have a very short half-life ~ 2 weeks [19,20]. Stressors such as exercise and starvation triggering mitophagy within 1−2 h in animal studies [35,36], mediated by 5' AMP-activated protein kinase (AMPKα1/α2/β2/γ1 or referred to as mitoAMPK) [37]. However, mitochondrial biogenesis, a process requiring over a week to double mitochondrial content [38], is insufficient to explain the rapid increases in mitochondrial markers observed in human skeletal muscle within 1 h post-exercise [39]. External mitochondrial donation from circulating bone marrow cells provides a more reasonable explanation for fast mitochondria gains in exercised muscle than mitochondrial biogenesis within contracting myofibers. Fresh mitochondrial delivery into myofibers by cell fusion from bone marrow-derived cells may facilitate rapid metabolic adaptation in response to autophagic stress. However, we speculate that the newly acquired mitochondria in repairing myofibers may not yet be fully functional, as their integration into the host cellular network requires nuclear DNA-encoded proteins for proper assembly of oxidative phosphorylation complexes [40].

### 4.4 Amino acids accelerate senolytic effect of resistance exercise

p16^Ink4a^ mRNA is a commonly used cellular senescence marker in humans [41], to assess senolytic effect of resistance exercise in the study. The senolytic effect of high-intensity interval exercise has been shown to peak around 3 h post-exercise and gradually subside within 24 h [42]. The reduction in muscle p16^Ink4a^ mRNA following such high-intensity exercise requires inflammation as an acute immune response [42]. In this study, a significant decrease in p16^Ink4a^ mRNA was observed 24 h after resistance exercise, suggesting an ongoing inflammation. Notably, amino acid supplementation led to a significant reduction in p16^Ink4a^ mRNA levels in human skeletal muscle immediately post-exercise. This response returned to baseline in 24 h, supporting an accelerated resolution of inflammation. Amino acids serve as the primary dietary nitrogen source essential for DNA synthesis and cell regeneration [4], which is at high demand after a muscle-damaging exercise. In contrast, the placebo group, which received an isocaloric carbohydrate supplement, exhibited a slower senolytic response. These findings highlight the critical role of dietary nitrogen in enhancing exercise-induced adaptations to inflammatory stress.

## 5 Limitations

One limitation in human studies is the difficulty of performing mitochondria-rich cell transplantation into skeletal muscle to restore aerobic respiration in cells with dysfunctional mitochondria, as demonstrated in animal studies. Mesenchymal stem cell transplantation has been shown to donate mitochondria to cells with defective or deleted mtDNA, thereby enhancing aerobic metabolism [32]. Furthermore, the selected time points (0 h and 24 h) may not adequately capture the early inflammatory signaling that likely occurred. Including an intermediate time point (e.g. 3 h post-exercise) would provide additional insight into the role of inflammation in mitochondrial gains for future studies. However, substantially greater mitochondria concentration in neutrophils than myofibers are very consistent across all muscle samples regardless a wide individuality.

Additionally, our findings in resistance exercise cannot be directly extrapolated to aerobic exercise responses. This is particularly relevant that aerobic exercise may induce different physiological adaptations and can also cause lung tissue stress in addition to skeletal muscle effects. Lungs are the most important competitor for immune cells [43] and bone marrow stem cells [44]. Furthermore, while neutrophils are mitochondria-rich cells, we could not rule out mitochondria donation from other types of cells (MPO-negative) to disrupted myofibers based on our immunofluorescence staining analysis.

## 6 Conclusion

The findings of the study indicate that pre-exercise amino acid supplementation accelerates the resolution of inflammation after resistance exercise, evidenced by a shorter period of neutrophil infiltration and reduced oxidative DNA damage in human skeletal muscle. These results provide a mechanistic basis for the use of pre-exercise amino acid supplementation to facilitate recovery from resistance exercise.

Furthermore, the markedly higher mitochondrial concentration observed in MPO⁺ neutrophils and other unidentified cell types, compared with the myofiber cytoplasm, along with the evident mitochondrial diffusion gradient from these cells to myofibers, suggests a plausible mechanism whereby contracting myofibers may replenish their mitochondria through the recruitment and integration of bone marrow-derived cells as part of a damage-induced regenerative response to resistance exercise.

## Consent for publication

Informed consent was obtained from all subjects involved in the study. No individual and personal data were shown in this manuscript.

## Data Availability

Data can be obtained upon reasonable request from the first author.
